# Intravenous Thrombolysis Preceding Mechanical Thrombectomy in Patients with Acute Ischemic Stroke Reduces the Inflammatory Response: Preliminary Results Based on Retrospective Analysis of Medical Documentation

**DOI:** 10.3390/jcm15072643

**Published:** 2026-03-31

**Authors:** Milena Świtońska, Agnieszka Rogalska, Alicja Szulc, Oliwia Jarosz, Magdalena Konieczna-Brazis, Łukasz Wołowiec, Wioletta Banaś, Magdalena Grigorief, Jacek Budzyński

**Affiliations:** 1 Department of Neurology and Clinical Neurophysiology, Ludwik Rydygier Collegium Medicum in Bydgoszcz, Nicolaus Copernicus University in Toruń, 85-168 Bydgoszcz, Poland; m.switonska@cm.umk.pl (M.Ś.); alicja.szulc@cm.umk.pl (A.S.); magda.brazis@cm.umk.pl (M.K.-B.); 2 Jan Biziel University Hospital No. 2 in Bydgoszcz, Ludwik Rydygier Collegium Medicum in Bydgoszcz, Nicolaus Copernicus University in Toruń, 85-168 Bydgoszcz, Polandlukasz.wolowiec@cm.umk.pl (Ł.W.); magdalena.grigorief@cm.umk.pl (M.G.); 3 Doctoral School of Medical and Health Sciences, Ludwik Rydygier Collegium Medicum in Bydgoszcz, Nicolaus Copernicus University in Toruń, 85-067 Bydgoszcz, Poland; 503671@doktorant.umk.pl; 4 Department of Cardiology and Clinical Pharmacology, Faculty of Health Sciences, Ludwik Rydygier Collegium Medicum in Bydgoszcz, Nicolaus Copernicus University in Toruń, 85-168 Bydgoszcz, Poland; 5 Department of Vascular and Internal Medicine, Ludwik Rydygier Collegium Medicum in Bydgoszcz, Nicolaus Copernicus University in Toruń, 85-168 Bydgoszcz, Poland

**Keywords:** acute ischemic stroke, inflammatory response, biomarkers, mechanical thrombectomy, intravenous thrombolysis

## Abstract

**Background:** Acute ischemic stroke (AIS) induces a severity of inflammatory response that varies depending on the individual and may depend on the type of reperfusion treatment used. The aim of this study was to compare values of inflammatory response indices between AIS patients treated with endovascular mechanical thrombectomy (EMT) only and those in whom EMT was preceded by intravenous thrombolysis (IVT). **Patients and methods:** Retrospective analysis of medical documentation of 2242 consecutive, real-world patients hospitalized in one center due to AIS between 1 January 2014 and 31 May 2025. Several single and composite inflammatory indices were analyzed. **Results:** Patients who underwent double reperfusion treatment (IVT + EMT) (n = 1201; 53.57%) had lower C-reactive protein (CRP)-to-albumin, CRP-to-lymphocyte, CRP-to-neutrophil, and CRP-to-platelet ratios; lower platelet-to-lymphocyte, platelet-to-albumin, and platelet-to-hemoglobin ratios; and a lower inflammatory burden and systemic inflammatory index than those who were treated only with EMT (n = 1041; 46.43%). Compared to patients treated only with EMT, those treated with IVT + EMT also had a shorter length of in-hospital stay, were less likely to be readmitted within 14 days of discharge, and were more likely to achieve a modified Rankin score of 0–1 at discharge. **Conclusions:** Patients with AIS treated with IVT + EMT may exert a lower inflammatory magnitude of response and better functional status at discharge than those treated with EMT only. Biomarkers of inflammatory response to AIS require further study to confirm their usefulness in AIS patients’ management and personalized qualification for reperfusion and non-reperfusion targeted treatment.

## 1. Introduction

Acute ischemic stroke (AIS) is the sudden local dysfunction of the central nervous system as a result of acute cerebral circulatory insufficiency, causing physical and mental dysfunction and leading to healthcare dependence and loss of employment and social functioning. In Poland, AIS occurs in more than 74 thousand people annually, of whom about 30% are younger than 65 years, and is the second leading cause of death and the most prevalent cause of loss of independent ambulation [1].

The current treatment for AIS caused by large artery occlusion (i.e., large artery atherosclerosis and cardio-embolism, according to the TOAST [Trial of ORG 10172 in Acute Stroke Treatment] classification) emphasizes time window-dependent vascular recanalization, predominantly relying on intravenous thrombolysis (IVT), endovascular mechanical thrombectomy (EMT), or their combined application, with reperfusion effectiveness rates amounting to 70–90% [2,3]. Unfortunately, even after technically successful reperfusion, only 30–50% of AIS patients achieve functional independence. One explanation of poor neurological outcomes despite successful target cerebral artery recanalization, known as futile recanalization, is that individually variable neuroinflammatory cascades triggered by both an ischemic event and post-reperfusion processes may exacerbate neuronal damage (known as a secondary injury or “second hit”) to a greater extent than that observed in patients treated medically [4,5]. Therefore, AIS was recently recognized as a thrombo-inflammatory disease [6].

During AIS, four stages of inflammatory phases typically start in the brain tissue: (a) widening of the capillaries; (b) increased microcirculation permeability in the blood–brain barrier and ligand expression (e.g., chemokines, selectins, integrins); (c) neutrophil attraction in tethering, rolling, arrest, adhesion, and crawling processes, followed by their extravasation and transmigration to the site of ischemic brain injury and the creation of neutrophil extracellular traps (NETs); and (d) systemic inflammatory response with leukocyte proliferation and blood coagulation activation [6,7,8]. Neutrophils migrate to necrotic brain tissue during the first few hours of ischemic stroke, and monocytes accumulate in the brain tissue during the first 24 h of AIS, whereas lymphocytes, as the ‘last’ leukocytes, with mainly anti-inflammatory and brain repair properties, reach the site of cerebral necrosis 24–48 h after a stroke [9,10]. At the site of brain necrosis, neutrophils: (a) secrete various enzymes (e.g., cytokines, chemokines, proteases, matrix metalloproteinase-9 [MMP-9]); and (b) influence blood coagulation through the stimulation of platelet aggregation, formation of platelet–leukocyte aggregates, secretion of tissue factor and the promotion of fibrin formation, and modulation of thrombus maturation, which determines long-term thrombus regression and recanalization.

The inflammatory response to AIS and neuroinflammation via the neurovascular network stimulates an increase in the concentration of blood inflammatory biomarkers, which are used as predictive factors of an unfavorable AIS course and patient prognosis. These biomarkers are as follows: C-reactive protein (CRP); high-sensitivity CRP (hs-CRP); interleukin-6 (IL-6); CRP-to-lymphocyte ratio; neutrophil-to-lymphocyte ratio (NLR); CRP × NLR, known as the Inflammatory Burden Index (IBI); CRP-to-albumin ratio; CRP-albumin-lymphocyte (CALLY) index; lymphocyte-to-monocyte ratio; platelet-to-lymphocyte ratio; systemic immune-inflammation index (SII); systemic inflammation response index (SIRI); Naples Prognostic Score (NPS); platelet-to-hemoglobin ratio; and monocyte-to-high-density lipoprotein ratio [2,4,5,6,7,8,9,10,11,12,13,14,15,16,17,18,19,20,21,22,23]. The worse prognosis for AIS patients with amplified local and systemic inflammatory response is explained by the secondary aggravation of brain injury due to neuroinflammation, coagulation process activation with venous thromboembolism, a decrease in thrombus reactivity to recombinant tissue plasminogen activator (rt-PA) (alteplase, recognized as a cause of low artery recanalization rates, which, among AIS patients, amounts to 20–46% [6]), and the promotion of cerebral artery thrombosis with re-occlusion of the previously opened vessel, the futile effect of reperfusion therapy (IVT, EMT), and reoccurrence of brain ischemia (delayed cerebral ischemia). Further factors in a poor prognosis for AIS patients include an increase in brain infarct volume, blood–brain barrier disruption, malignant brain edema, stroke hemorrhagic transformation, atherosclerotic plaque instability, and systemic complications (e.g., pneumonia, urinary tract infection, pressure wounds, and atrial fibrillation) [6,8,9,11,14,18,21,24,25].

Some studies show better clinical outcomes among AIS patients treated with “double reperfusion therapy” (IVT preceding EMT) than for those treated with IVT or EMT alone [10,26,27,28,29]. These observations are explained by avoiding treatment delay in patients qualified for EMT, more effective removal of the thrombus from occluded vessels, restoring the forward blood flow in large and small vessels and in microcirculation (lowering the probability of the “no-reflow” phenomenon), and reduction of brain injury, among other things, due to shorter duration of cerebral ischemia [11].

Therefore, we hypothesized that AIS patients treated with IVT preceding EMT would present a lower level of neuroinflammation, systemic inflammation, and oxidative stress response, and that this would promote the recovery of neurological function and reduce the neurological impairment when compared with those patients who underwent EMT only. Confirmation of this hypothesis could be the premise for studies on the combination of reperfusion AIS therapy with personalized treatment on the basis of the predictive values of the proinflammatory response indices observed. Non-reperfusion targeted AIS therapies investigated until now consisted of: (a) neuroinflammation-targeted substances; for example, methylprednisolone, colchicine, canakinumab, immuno-nutrition with omega-3 fatty acids, food-derived exosomes, and inflammasome modulating substances; and (b) neutrophil-targeted substances, such as interferon-ß, targeting NETs with DNase 1, lipoxygenase and leukotriene pathway therapies, along with anti-interleukin-1, selective suppression of peptidyl arginine deiminase 4 (PAD4), P-selectin glycoprotein ligand-1 (PSGL-1), and CD40-ligand [6,8,30,31,32,33,34,35,36,37,38]. However, it is not known whether AIS patients on IVT may, due to the lower stimulation of inflammatory response, be recognized as potential candidates for such additional treatment. Therefore, we performed an analysis of medical documentation of AIS patients treated with EMT in a single neurological center at a university hospital to compare the values of laboratory indices of inflammatory response between those in whom EMT was and was not preceded by IVT.

## 2. Materials and Methods

### 2.1. Patients

We analyzed medical documentation of 2242 consecutive patients treated with EMT in one university hospital due to AIS between 1 January 2014 and 31 May 2025, with the intention of achieving cerebral reperfusion. The inclusion criteria were: acute ischemic stroke (code I63, according to the International Classification of Diseases, Tenth Revision [ICD-10]) qualified for treatment with EMT, preceded or not by IVT, with rt-PA (alteplase (Boehringer Ingelheim, Ingelheim am Rhein, Germany): 0.9 mg/kg of body weight; maximum 90 mg per one hour); and aged 18 or more years of age. The exclusion criteria were: transient ischemic attack; hemorrhagic stroke; clinical evidence of infection at admission; and history of leukemia. AIS was diagnosed by an experienced neurologist, and cerebral artery occlusion was confirmed using computed tomography angiography. Comorbidities and atherosclerotic cardiovascular disease risk factors (e.g., hypertension, diabetes mellitus [DM], dyslipidemia, smoking habit, coronary artery disease, chronic cardiac failure, atrial fibrillation [AF], and cancer) were identified on the basis of the data available from interviews contained in the medical documentation. EMT was performed by an experienced, certificated interventional radiologist. EMT was recognized as successful when angiographic appearances of the treated occluded vessel and the distal branches after final passage in EMT amounted to a 2b, 2c or 3 score in the modified Thrombolysis in Cerebral Infarction (mTICI) scale. Additional patient management, pharmacotherapy and physiotherapy were applied in accordance with the current Polish recommendations.

### 2.2. Methods

This study relied on real-world retrospective analysis of the electronic medical documentation of all consecutive patients hospitalized in the Neurology Department of a university hospital due to AIS. Patients were identified on the basis of the primary diagnosis at discharge (code I63, according to the ICD-10) between 1 January 2014 and 31 May 2025. We obtained clinical and laboratory data measured using standard methods at a certified central hospital laboratory (the first measurement was taken during hospitalization, after intervention). The data were as follows: blood smear morphology of white blood cells (leukocytes), and a profile of blood CRP; albumin; thyroid-stimulating hormone; total, high-density lipoprotein (HDL), and low-density lipoprotein (LDL) cholesterol; and triglycerides, glucose, HbA1c, and creatinine concentrations. Not all biochemical determinations were available for all patients.

The following single parameters were used as biomarkers of inflammatory response to AIS: blood CRP, IL-6, and albumin concentrations; leukocyte and platelet absolute counts; and leukocyte differentials: neutrophils, lymphocytes, and monocytes in absolute counts and percentages in relation to total leukocyte count. We also calculated composite inflammatory response indices, such as: CRP-to-albumin ratio (CAR), CRP-to-lymphocyte ratio (CLR), CRP-to-neutrophil ratio, and CRP-to-monocyte ratio; CRP-to-platelet ratio; CRP-to-HDL-cholesterol ratio [22]; neutrophil-to-lymphocyte ratio (NLR), neutrophil-to-platelet ratio (NPR), and lymphocyte-to-monocyte ratio (LMR); platelet-to-lymphocyte ratio (PLR), platelet-to-albumin ratio, and platelet-to-hemoglobin ratio (PHR) [23]; lymphocyte-to-albumin ratio; and monocyte-to-HDL ratio (MHR) [39]. IBI was calculated according to the following formula: IBI = CRP (mg/dL) × NLR [21]; CALLY index was calculated using the formula: CALLY = [albumin (mg/dL) × lymphocytes (G/L)]/[CRP (mg/dL) × 10] [5]; HLAN index was calculated according to the formula: HLAN = hemoglobin (g/L) × lymphocytes (G/L) × albumin (g/L)/neutrophils (G/L)/100; HALP index was calculated using the following: HALP = hemoglobin (g/L) × albumin (g/L) × lymphocytes (G/L)/platelets (G/L); SIRI was calculated according to the formula: SIRI = neutrophils × monocytes/lymphocytes; SII was calculated according to the formula: SII = neutrophil count × PLR [13]; and NPS was calculated according to scored cut-offs for albumin, total cholesterol, NLR, and LMR [4].

### 2.3. Outcomes Measured

The following outcomes were included in the analysis:

- values of the single and composite inflammatory indices referred to above;
- in-hospital all-cause mortality; readmission within 14, 30, and 365 days of discharge; length of in-hospital stay (LOS); scores and their changes (delta) between discharge and admission using the following neurological patient disability and dependence scales: National Institutes of Health Stroke Scale (NIHSS), modified Rankin Scale (mRS), and a score on the mRS < 2 (0–1) at discharge.

### 2.4. Bioethics

This study was a retrospective analysis of anonymized medical documentation of consecutive patients with AIS hospitalized in one center, a university hospital, within the past ten years. This study was not registered as a clinical trial. The investigation was conducted in compliance with the Declaration of Helsinki for medical research. The investigation protocol was approved by the local, independent Ethical Committee on human experimentation (Bioethical Committee of Nicolaus Copernicus University in Toruń by Ludwik Rydygier Collegium Medicum in Bydgoszcz; approval No. KB 376/2025 on 25 June 2025). According to Polish regulations, including the ruling issued by the Bioethics Committee, patient informed consent was not required for retrospective analyses of anonymized medical documentation. Furthermore, the study cohort consisted of patients with AIS, for whom obtaining informed consent would have been impracticable given the cognitive impairments associated with this clinical condition, and ten years after stroke occurrence, the majority of patients analyzed had passed away.

### 2.5. Statistics

Statistical analysis was conducted using the licensed version of the statistical software Statistica, version 13.3, developed by Tibco Software, Inc. 2017 (Palo Alto, CA 94304, USA, San Ramon, CA 94583, USA). The normal distribution of the study variables was checked using the Kolmogorov–Smirnov test. The results are presented as the mean ± standard deviation, n, %, and median, and interquartile range. The statistical significance of differences between groups was verified using the Student’s *t*-test, Mann–Whitney U test, and Chi^2^ test. Multiple regression was used to determine factors influencing the values of the respective inflammatory response indices. The following independent variables were introduced: patient’s age; gender; history of comorbidities (e.g., DM, AF, hypertension, chronic coronary syndrome, chronic kidney disease); IVT; body mass index (BMI); Glasgow Coma Scale (GCS), mRS, and Nutrition Risk Screening 2002 (NRS-2002) scores at admission; leukocyte count; blood concentrations of hemoglobin, LDL cholesterol, HbA1c, and creatinine; and international normalized ratio (INR) and activated partial thromboplastin time (aPTT) results. Receiver Operating Characteristic (ROC) analysis was also used to determine cut-offs of inflammatory response indices predicting occurrence of outcome measures mentioned above. Logistic regression was used to determine factors influencing IVT use, reperfusion success in EMT, all-cause in-hospital mortality, readmission and mRS score < 2, as well as for calculation of HR associated with binary values of biomarkers studied, and it was based on cut-offs obtained in ROC analysis. The statistical significance level was set at a *p*-value of <0.05.

## 3. Results

We examined the medical documentation of 2242 consecutive AIS patients treated with EMT. When compared to patients only treated endovascularly (n = 1041, 46.43%), those in whom EMT was preceded by IVT (n = 1201, 53.57%; i.e., by double cerebral reperfusion therapy), despite similar probability to reach reperfusion success in EMT (measured in TICI score which was better than 2b, which means: “complete filling of all of the expected vascular territory is visualized but the filling is slower than normal”), had a statistically significantly shorter LOS and a shorter delay between admission to the emergency department and admission to the neurological ward (Table 1). Compared to patients treated with EMT only, those treated with IVT and EMT were less likely to be readmitted within 14 days of discharge and had a lower score following an NRS-2002 survey. Moreover, patients on double cerebral reperfusion therapy had statistically significantly higher Barthel and NIHSS scores at admission and lower mRS scores at admission and discharge (Table 1). Patients on double reperfusion therapy were also more likely to achieve mRS scores of 0–1 at discharge than patients treated with EMT only.

With regard to laboratory determinations, compared to patients treated with EMT only, those in whom EMT was preceded by IVT had a lower platelet count, lower INR values, and lower blood CRP concentrations, as well as a higher blood LDL cholesterol concentration (Table 2).

For indices of inflammatory response (Table 3), compared to patients treated with EMT only, those who underwent double reperfusion treatment had lower values for the following: CRP-to-albumin, CRP-to-lymphocyte, CRP-to-monocyte, CRP-to-neutrophil, and CRP-to-platelet ratios; lower IBI (CRP × NLR); lower platelet-to-albumin, platelet-to-lymphocyte, and platelet-to-hemoglobin ratios; lower neutrophil-to-albumin and monocyte-to-albumin ratios; and a lower SII (Table 3). For these parameters, size effects were also statistically significant. We did not find significant differences between the groups studied with regard to the other composite biomarkers used in AIS patients’ risk stratification, such as the CALLY index and the monocyte-to-HDL ratio.

In multifactorial analysis, using a multiple regression method, we found that the use of IVT was a significant and independent factor negatively influencing the variance in CRP-to-albumin, CRP-to-lymphocyte, CRP-to-neutrophil, and CRP-to-monocyte ratios; platelet-to-lymphocyte, platelet-to-albumin, and platelet-to-hemoglobin ratios; IBI; and monocyte-to-albumin ratio (data not presented in detail).

In the ROC analysis, we did not find any statistically significant relationships between values of inflammatory response biomarkers studied and achieving successful reperfusion in EMT and 14-day readmission after discharge. Regarding all-cause in-hospital mortality, only CALLY with a cut-off amounting to 3.47 reached a weak performance level and was associated with 57% reduction of in-hospital death (Table 4). Whereas, in relation to achieving an mRS score below 2 at discharge, even the four biomarkers studied reached a level of accepted performance (AUC > 0.700). They were as follows: NLR, SII, SIRI, and IBI (Table 4). Patients having values of the respective composed biomarkers of inflammatory response amounting to equal or above the cut-offs obtained in ROC analysis were 2–4 times less likely to be functionally independent at discharge, as expressed by hazard ratio (HR) values lower than 1.

## 4. Discussion

In this study, we revealed, among other things, shorter LOS, lower risk of 14-day readmission, and better functional status at discharge for AIS patients treated with double reperfusion therapy than for those treated with EMT only (Table 1). These better clinical outcomes were associated with lower values of systemic inflammatory response indices in patients with AIS treated with EMT preceded by IVT in comparison to those treated with EMT only (Table 2 and Table 3). In multiple regression, IVT was found to be an independent and significant factor determining variance in the majority of inflammatory response indices studied. Lower values of biomarkers of inflammatory response were also associated with lower risk of all-cause in-hospital mortality, as well as with higher probability of functional independence at discharge (Table 4).

The results of this study corroborate research showing an advantage of double reperfusion AIS treatment (IVT + EMT) over EMT therapy alone with regard to patients’ functional status scores (mRS) [9,10,11,25,27,40,41]. Data provided by other authors revealed proinflammatory activity of alteplase (rt-PA) through neutrophil recruitment; upregulation of adhesion molecules in the endothelium; activation of plasma kinins, the complement system, and extrinsic pathway of coagulation activation with simultaneous inhibition of clot lysis by rt-PA due to a sustained increase in plasminogen activator inhibitor type-1 (PAI-1) induced by inflammatory mediators [13,42,43]; and potential rt-PA activity causing brain–blood barrier disruption [44]. Our study showed that, compared to EMT alone, IVT preceding EMT in the therapy of AIS was associated with a lower blood level of inflammatory response indices, expressed by lower leukocyte and platelet counts; lower blood CRP concentration; lower ratios of CRP to albumin, neutrocytes, lymphocytes, monocytes, and platelets; lower IBI; a lower ratio of platelets to lymphocytes, neutrophils, and albumin; and lower SII (Table 3). Such observations corroborate a study by Li et al. [2], who showed a lower inflammatory response in AIS patients treated with IVT + EMT compared to those treated with EMT only. Better AIS treatment outcomes were associated in our study (Table 4) with lower values of inflammatory response biomarkers (with regard to all-cause mortality (CALLY index) and independence (e.g., CRP-platelet ratio, NLR, SII, SIRI, IBI), and in the other studies were also associated with single-nucleotide polymorphism in CRP [45], lower values of CRP [18,46], lower hs-CRP [14], lower CLR [16,43], lower NLR and higher LMR [2,10], lower CAR and neutrophil and lymphocyte count and NLR [22], and lower SII [13,24]. Moreover, among patients with AIS, CLR was a recognized biomarker of disruption to the blood–brain barrier due to kinin system activation and the easier migration of respective leukocyte differentials and platelets to the sites of brain necrosis. In addition, CLR links the inflammatory response with nutritional status, and a high CLR value is recognized as a factor influencing AIS patients’ prognosis, indicating deterioration and poor outcomes [22]. In a study by Mo et al. [21], elevated IBI was significantly associated with a more than four-fold increase in all-cause mortality, as well as with higher stroke recurrence, dependency, and poorer functional outcome, as in our study (Table 4). In Pan et al. [5], a higher CALLY index was associated with a lower risk of AIS hemorrhagic transformation, and its predictive value surpassed that of other known scores (e.g., Hemorrhage Risk Stratification, Hemorrhagic Transformation Index, Stroke Prognostication using Age and NIHSS, Hemorrhage After Thrombolysis, Symptomatic Intracranial Hemorrhage after Stroke Thrombolysis, and GWTG-Stroke sICH risk scores). In Koldborg et al. [18], CRP level at admission correlated with LOS. Wu et al. [8] reported that SII is also closely associated with long-term prognosis in AIS patients.

The following are proposed as potential pathomechanisms for explaining a lower inflammatory response with dual than with single reperfusion AIS therapy: a more gradual and slower cerebral reperfusion process in patients with IVT and better thrombus preparation for EMT, making possible total clot removal and the restoration of the forward flow in blood vessels, not only in large arteries but also in small cerebral arteries and in microcirculation [11,17,43,47]. The potential for alteplase in the reduction of oxidative stress in brain tissue due to the induction of a lower level of ischemia-reperfusion processes, and milder systemic inflammatory response to ischemic brain injury in AIS patients on IVT has also been reported [2,3,42]. Moreover, the therapeutic window for IVT is narrow and concerns only the first 4.5 h following the beginning of AIS symptoms; EMT can even be used up to 24 h after, so reperfusion can occur earlier in patients treated with IVT. When reperfusion occurs later, the proportion of ischemic penumbra to brain tissue necrosis decreases, which may increase the intensity of ischemia-reperfusion processes, inflammatory responses, disruption of the blood–brain barrier, and deterioration in microcirculation, leading to a worsening in the patient’s functional status [17,47]. On the other hand, the efficacy of IVT in AIS treatment depends on thrombus size, its composition (fibrin, platelets, NETs, etc.), its location, and earlier collateral circulation development as a potential pathway for oxygen, glucose, and rt-PA being provided to the ischemic penumbra site. Therefore, these features should be taken into account as potential confounding factors in the outcomes of reperfusion therapy [17,42]. Today, these factors can be investigated in detail because EMT offers the possibility of collecting clots from cerebral arteries and studying their structure, and arteriography can show cerebral collateral status as a potential substrate for compensatory circulation development.

In our study, we did not confirm the significance of common inflammatory response indices (such as NLR, NPR, LMR, and SIRI) [2,4,13,39,47] as discriminating factors of neuroinflammation severity between AIS patients treated with double and single reperfusion therapy (Table 3). However, we revealed significant differences between the groups studied with regard to IBI values (Table 3 and Table 4), which offers a more comprehensive inflammatory response assessment by integrating CRP levels with NLR [21]. We also revealed the predictive power of CPR, NLR, NPR, SII, SIRI, and IBI for being functionally independent at discharge (Table 4). SIRI was also found to have no predictive value among AIS patients in a study by Wang et al. [13].

The practical implication of the results of our study is the necessity to perform further studies to evaluate the pathomechanism of the anti-inflammatory activity of IVT in patients with AIS, evaluate the predictive power of inflammatory biomarkers in studies designed as time-anchored, and assess the role of non-reperfusion targeted agents, as described in the introduction, especially in patients who underwent EMT only, as suggested by the results of this study.

As with most authors, we could not avoid some shortcomings that could influence the strength of the conclusions that were based on our results. Firstly, the retrospective and one-center study design with only medical documentation analysis should be considered the main limitation. Nonetheless, the study was performed on a large sample size of more than two thousand subjects. Secondly, the laboratory data used as the basis for comparisons made in Table 3 were not available for all patients who underwent EMT during the period analyzed; however, respective missing-labs did not exceed 5% of patients enrolled in the analysis. Taking this into account, we performed an available-case analysis (pairwise deletion) instead of listwise deletion. Thirdly, we did not analyze serial measurements of inflammatory response indices. Several studies further indicated that dynamic changes in inflammatory indices within 24–48 h after IVT provided stronger prognostic discrimination than baseline measurements, underscoring the clinical value of early immune monitoring during the acute phase of stroke and, therefore, a single determination at admission might lead to bias [9,20]. This confirms the need for further time-anchored laboratory determination, relative, for example, to start with IVT, first pass, final reperfusion after EMT, etc. Fourthly, we performed multifactorial analysis of factors influencing both inflammatory response biomarker values and their relationship to outcome measured (e.g., in-hospital all-cause mortality, readmission, achieving mRS score < 2 at discharge); however, we obtained either equations statistically not significant or equations with a low value of R^2^ coefficient. On the other hand, we revealed the prognostic power of six biomarkers (Table 4) with regard to achieving patient functional independence at discharge.

Nevertheless, our study introduces some novelty to the knowledge concerning acute inflammatory responses in patients with AIS treated with EMT only. The strong point of this work is that, to the best of our knowledge, after Zeng et al. [29], ours is only the second study worldwide to assess the difference between inflammatory response indices between patients with AIS for whom double and single reperfusion therapy was applied. Our work may thus be an important contribution to knowledge concerning the personalization of AIS non-reperfusion targeted treatment based on the inter-individual variability of inflammatory response to brain injury. Moreover, in addition to common inflammatory biomarkers (e.g., NLR, PLR, and SII), we also evaluated new composite biomarkers (e.g., CALLY index, PHR, MHR, and IBI), which integrate cellular (e.g., NLR) and humoral (e.g., CRP, albumin) inflammatory responses and are recognized as surpassing other biomarkers in prognostic accuracy for patients with AIS [21].

## 5. Conclusions

Taking into account the study limitations enumerated above and the need for careful interpretation of the results obtained, we suggest that patients with AIS treated with double reperfusion therapy (IVT + EMT) had a lower magnitude of inflammatory response to stroke than patients treated with EMT only. Patients in whom EMT was preceded by IVT were more likely to be discharged with an mRS score of < 2, and lower values of biomarkers of inflammatory response exert acceptable power to predict functional independence in AIS patients. Nonetheless, biomarkers of inflammatory response in patients with AIS need further study with time-anchored biomarker analyses strictly relative to IVT bolus and/or final reperfusion to confirm the role of individual inflammatory response to brain injury in the course of AIS and usefulness of these biomarkers in AIS patients’ management in both the pre- and post-recanalization periods to optimize and personalize patients’ selection for respective reperfusion and non-reperfusion targeted treatment, as well as postoperative management and prognosis personalization.

## Figures and Tables

**Table 1:** Comparison of clinical characteristics of patients with acute ischemic stroke between those treated with mechanical thrombectomy preceded by intravenous thrombolysis and those who were treated with mechanical thrombectomy only (N = 2242).

Parameter	Thrombectomy + Thrombolysis (n = 1201; 53.57%)	Thrombectomy Only (n = 1041; 46.43%)	*p*
Age (years)	71.71 ± 13.20	72.21 ± 12.88	0.366
Male gender (n, %)	573 (47.71)	501 (48.13)	0.999
Length of in-hospital stay (days)	7; 0–12	8; 0–13	0.101
Length of hospitalization in neurology department (days)	6; 0–11	7; 0–12	0.327
Delay between admission to emergency department and neurology ward (hours)	1.01 ± 0.06	1.02 ± 0.12	<0.001
All-cause in-hospital death (n, %)	246 (20.48)	238 (22.86)	0.172
Readmission within 14 days of discharge (n, %)	15 (1.25)	28 (2.69)	0.013
Readmission within 30 days of discharge (n, %)	42 (3.50)	49 (4.71)	0.148
Readmission within 365 days of discharge (n, %)	136 (11.32)	138 (13.26)	0.257
BMI (kg/m^2^)	28.25 ± 6.02	28.21 ± 6.29	0.969
Ideal body mass (%)	129.28 ± 27.85	129.54 ± 29.12	0.949
NRS-2002 score at admission	2.49 ± 0.70	2.56 ± 0.83	0.020
GCS score at admission	12.58 ± 3.16	12.42 ± 3.32	0.312
Barthel Index (score) at admission	10; 0–60	5; 0–45	0.004
NIHSS score at admission	15; 11–19	14; 10–19	0.011
NIHSS score at discharge	11; 5–17	10; 5–16	0.603
mRS score at admission	4; 2–5	4; 3–5	<0.001
mRS score at discharge	2; 1–4	3; 1–4	0.010
mRS 0–1 at discharge (n, %)	502 (41.80)	317 (30.45)	0.019
Norton Scale (score) at admission	9.97 ± 3.28	9.92 ± 3.27	0.741
VES-13 (score) at admission	4.70 ± 3.47	5.05 ± 3.29	0.093
MFS scale (score) at admission	35.99 ± 17.38	37.20 ± 16.79	0.159
mTICI ≥ 2b (reperfusion success; n, %)	975 (96.53)	818 (95.45)	0.231

Abbreviations: BMI = body mass index; GCS = Glasgow Coma Scale; MFS = Morse Fall Scale; mRS = modified Rankin Scale; NIHSS = National Institutes of Health Stroke Scale; NRS-2002 = Nutrition Risk Screening 2002; mTICI = The modified Thrombolysis in Cerebral Infarction score; VES-13 = Vulnerable Elders Survey. Data presented as mean ± standard deviation, and median; IQR, depending on variable distribution.

**Table 2:** Comparison of laboratory determinations of patients with acute ischemic stroke between those treated with mechanical thrombectomy preceded by intravenous thrombolysis and those who were treated with mechanical thrombectomy only (N = 2242).

Parameter	Thrombectomy + Thrombolysis (n = 1201; 53.57%)	Thrombectomy Only (n = 1041; 46.43%)	*p*
Red blood cells (T/L)	4.26 ± 0.59	4.27 ± 0.62	0.671
Hemoglobin (g/L)	13.04 ± 1.82	12.89 ± 2.00	0.136
Hematocrit (%)	38.32 ± 5.09	38.23 ± 5.49	0.714
Leukocytes (G/L)	11.10 ± 5.34	11.10 ± 4.83	0.99
Platelet count (G/L)	221.27 ± 69.36	236.22 ± 90.35	<0.001
Neutrophil count (G/L)	7.92; 5.60–10.76	7.96; 5.57–11.12	0.997
Lymphocytes (G/L)	1.49; 0.99–1.96	1.42; 0.99–2.00	0.606
Monocytes (G/L)	0.77; 0.57–1.01	0.77; 0.58–1.03	0.595
Eosinophils (G/L)	0.04; 0.00–0.12	0.05; 0.01–0.15	0.95
Total cholesterol (mg/dL)	145.84 ± 41.26	140.19 ± 40.28	0.208
HDL cholesterol (mg/dL)	44.19 ± 14.05	43.10 ± 15.38	0.668
Non-HDL cholesterol (mg/dL)	112.54 ± 46.53	99.97 ± 36.22	0.102
LDL cholesterol (mg/dL)	105.92 ± 45.06	94.79 ± 42.53	<0.001
Triglycerides (mg/dL)	119.08 ± 68.36	116.57 ± 54.11	0.663
Glucose (mg/dL)	136.56 ± 52.53	137.68 ± 48.78	0.775
HbA1c (%)	6.13 ± 1.37	6.21 ± 1.65	0.667
Creatinine (mg/dL)	0.99 ± 0.37	1.17 ± 0.70	0.172
Albumin (g/L)	3.32 ± 0.52	3.26 ± 0.51	0.234
CRP (mg/dL)	5.4; 2.3–15.4	7.9; 2.6–20.2	<0.001
aPTT (seconds)	28.78 ± 12.70	29.44 ± 12.89	0.31
INR	1.09 ± 0.24	1.15 ± 0.39	<0.001
Uric acid (mg/dL)	5.76 ± 1.80	5.77 ± 2.27	0.952
TSH (mU/L)	0.99; 0.58–1.80	1.06; 0.60–1.83	0.418

Abbreviations: aPTT = activated partial thromboplastin time; CRP = C-reactive protein; HDL = high-density lipoprotein; INR = international normalized ratio; LDL = low-density lipoprotein; TSH = thyroid-stimulating hormone. Data presented as mean ± standard deviation, and median; IQR, depending on variable distribution.

**Table 3:** Comparison of inflammatory response indices of patients with acute ischemic stroke between those treated with mechanical thrombectomy preceded by intravenous thrombolysis and those who were treated with mechanical thrombectomy only (N = 2242).

Index	Thrombectomy + Thrombolysis (n = 1201; 53.57%)	Thrombectomy Only (n = 1041; 46.43%)	*p*	Effect Size 95% CI
CRP-to-albumin ratio	1.96; 0.76–4.70	3.36; 0.89–10.14	<0.001	−5.18; −8.24–−2.11
CRP-to-lymphocyte ratio	3.77; 1.33–11.05	5.42; 1.76–18.22	<0.001	−9.65; −14.99–−4.31
CRP-albumin-lymphocyte index (CALLY index)	6.23; 2.50–17.20	3.82; 1.12–13.66	0.926	−0.54; −12.09–11.00
CRP-to-neutrophil ratio	0.70; 0.29–1.66	1.04; 0.38–2.27	0.003	−1.34; −2.23–−0.45
CRP-to-monocyte ratio	7.54; 2.77–19.64	10.34; 3.59–28.07	<0.001	−18.05; −27.85–−8.24
CRP-to-platelet ratio	0.03; 0.01–0.07	0.03; 0.01–0.08	0.012	−0.02; −0.04–−0.01
CRP-to-HDL cholesterol ratio	0.07; 0.03–0.20	0.10; 0.04–0.30	0.138	−0.37; −0.85–0.12
Neutrophil-to-lymphocyte ratio (NLR)	5.23; 3.18–9.05	5.62; 3.11–9.77	0.506	−0.28; −1.12–0.55
IBI (CRP × NLR)	29.35; 9.17–110.50	44.86; 12.22–161.63	<0.001	−108.1; −169.0–−47.3
Neutrophil-to-platelet ratio	51.34; 34.79–80.57	54.22; 34.04–83.27	0.351	−2,70; −8.39–2.99
Platelet-to-lymphocyte ratio	146.30; 104.41–209.84	164.63; 106.47–236.73	0.004	−29.15; −48.98–−9.32
Platelet-to-albumin ratio	66.22; 51.98–81.33	68.95; 50.29–97.20	0.038	−8.21; −15.96–−0.46
Platelet-to-hemoglobin ratio	16.21; 13.33–19.71	16.75; 13.46–22.24	<0.001	−1.64; −2.47–−0.82
Lymphocyte-to-albumin ratio	0.37; 0.25–0.49	0.42; 0.28–0.58	0.168	−0.12; −0.30–0.05
Neutrophil-to-albumin ratio	2.68; 1.76–3.60	2.75; 2.04–4.15	0.014	−0.50; −0.89–−0.10
Lymphocyte-to-monocyte ratio	1.94; 1.24–2.78	1.82; 1.23–2.81	0.96	−0.02; −0.98–0.93
Monocyte-to-HDL cholesterol ratio	0.02; 0.01–0.02	0.02; 0.01–0.03	0.291	−0.00; −0.01–0.00
Monocyte-to-albumin ratio	0.23; 0.16–0.33	0.27; 0.19–0.35	0.049	−0.05; −0.10–−0.00
HLAN index	6.08; 3.51–9.89	5.53; 3.19–11.27	0.296	−2.50; −7.19–2.20
HALP index	23.88; 15.53–36.00	22.07; 14.99–40.72	0.132	−13.45; −30.95–4.06
SII	1110.57; 663.45–2022.76	1279.45; 657.56–2316.59	0.028	−277.8; −526.2–−29.4
SIRI	4.03; 2.16–7.66	4.35; 2.10–8.64	0.422	−0.41; −1.43–0.60
NPS	4.00; 3.00–4.00	4.00; 3.00–4.00	0.222	−0.10; −0.27–0.06

Abbreviations: CRP = C-reactive protein; HALP = hemoglobin, albumin, lymphocytes, platelets; HDL = high-density lipoprotein; HLAN = hemoglobin, lymphocytes, albumin, neutrophils; NPS = Naples Prognostic Score; SII = systemic immune-inflammation index; SIRI = systemic inflammation response index. Notes: Data presented as median; IQR; Mann–Whitney U test.

**Table 4:** Parameters of ROC analysis for selected, statistically and clinically significant inflammatory response biomarkers with regard to their predictive value.

Parameter	Cut-Off	AUC; 95%CI;	*p*	HR; 95%CI, *p* for Cut-Off
All-cause in-hospital mortality				
CRP-albumin-lymphocyte index (CALLY index)	3.47	0.646; 0.611–0.81	<0.001	0.43; 0.26–0.68; <0.001
mRS score < 2 at discharge				
CRP-to-platelet ratio (CPR)	0.022	0.675; 0.640–0.709	<0.001	0.30; 0.19–0.44; <0.001
Neutrophil-to-lymphocyte ratio (NLR)	5.06	0.707; 0.655–0.759	<0.001	0.37; 0.18–0.75; <0.01
Neutrophil-to-platelet ratio (NPR)	30.05	0.621; 0.566–0.677	<0.01	0.48; 0.22–1.05; 0.68
SII	752.42	0.703; 0.651–0.755	<0.001	0.31; 0.15–0.64; <0.002
SIRI	3.05	0.700; 0.644–0.755	<0.001	0.25; 0.12–0.52; <0.001
IBI (CRP × NLR)	16.85	0.705; 0.653–0.757	<0.001	0.32; 0.15–0.67; <0.002

Abbreviations: SII = systemic immune-inflammation index; SIRI = systemic inflammation response index; cut-off value was calculated as Youden index; HR (hazard ratio) was calculated using logistic regression.

## Data Availability

The original contributions presented in this study are included in the article. The anonymized data supporting the findings of this study may be available from the corresponding authors upon reasonable request, subject to institutional data-sharing policies. The data are not publicly available due to privacy and ethical restrictions.
